# Epigenetic Deregulation of Transposable Elements Links Developmental Processes and Tumorigenesis

**DOI:** 10.3390/ijms27062690

**Published:** 2026-03-16

**Authors:** Chiemi Lynch-Sutherland, Peter Stockwell, Aniruddha Chatterjee, Michael R. Eccles, Erin Macaulay

**Affiliations:** 1 Department of Pathology and Molecular Medicine, Dunedin School of Medicine, University of Otago, P.O. Box 56, Dunedin 9054, New Zealand; clynch-sutherland@cmri.org.au (C.L.-S.); peter.stockwell@otago.ac.nz (P.S.); aniruddha.chatterjee@otago.ac.nz (A.C.); erin.macaulay@otago.ac.nz (E.M.); 2 Children’s Medical Research Institute, Westmead, NSW 2145, Australia; 3 Kids Neuroscience Centre, Kids Research, The Children’s Hospital at Westmead, Westmead, NSW 2145, Australia

**Keywords:** transposable element, DNA methylation, epigenetic regulation, embryonic development, cancer

## Abstract

Dedifferentiation—the acquisition of an early developmental state—is a hallmark of cancer. However, the underlying mechanisms that lead to cancer-associated dedifferentiation are not fully understood. Transposable elements (TEs) are becoming increasingly recognised as important regulators of development and disease. The recruitment of TE sequences has played an important role in placental evolution, and TE-derived genes play critical roles in placental development. Although important biological differences exist between tumours and the placenta, the placenta shares certain features with tumours, including the capacity to invade surrounding tissue and modulate the maternal immune response. In this regard, TEs have been implicated in cancer development, and are documented to contribute to oncogenesis through multiple different mechanisms. Moreover, cancers reacquire an epigenetic landscape, which is reflective of early development, and which corresponds to increased phenotypic plasticity, including facilitating the activation of early developmental genes. Many cancers can repurpose developmental genes, including TE-associated genes, which may contribute to pathways involved in invasion and metastasis. Determining whether TE activation is a consequence of broader epigenetic reprogramming or actively contributes to dedifferentiation will be important for understanding cancer biology and may facilitate improvements in cancer diagnosis and treatment.

## 1. Introduction: Transposable Elements and Epigenetic Regulation

Human development is an intricately regulated process that has evolved over millions of years. Epigenetic regulation is central to development, shaping the landscape that guides an individual totipotent cell to divide, eventually becoming a complete organism composed of diverse, specialised cell types contributing to an organism’s function. Precise spatial and temporal regulation of genes and pathways is essential for healthy development and for directing cell fate specification. As a result, each cell type has a unique epigenetic profile that drives its characteristic gene expression pattern, which gives rise to its phenotype and function.

Transposable elements (TEs) are repetitive DNA sequences that occupy a substantial proportion of mammalian genomes and comprise multiple classes, including DNA transposons, long-terminal repeat (LTR) retrotransposons, and non-LTR retrotransposons such as long interspersed nuclear elements (LINEs) and short interspersed nuclear elements (SINEs). Their ability to mobilise within the genome has contributed to TE sequences comprising ~45% of the human genome as repetitive sequences [1,2]. Multiple layers of regulation have evolved to suppress TE activity, including DNA methylation and repressive histone modifications such as trimethylation of histone H3 lysine 9 (H3K9me3). In particular, KRAB-associated protein 1 (KAP1)-mediated recruitment of the histone-lysine N-methyltransferase, SETDB1, facilitates the deposition of H3K9me3 at specific TE families, contributing to stable transcriptional silencing [3]. However, broad developmentally regulated changes in DNA methylation levels that occur during embryogenesis can facilitate TE reactivation and expression in early developmental tissues. Similarly, cancers share certain properties with some early developmental tissues, although key differences exist across species and tissue types. TE reactivation and expression are also frequently observed in cancer tissues.

This review summarises three different viewpoints, each of which is valid for different subsets of TEs and their contribution towards normal development and disease: (1) TEs act as passive passengers, and are normally silenced, but may become reactivated during the process of normal placental development and somatic tissue ageing, as well as in several disease states by global hypomethylation, although their contribution to development, ageing and disease is minimal. (2) TEs act as developmentally selected regulatory modules; it is critical they become activated during foetal and placental development, during which they possess essential functions to facilitate normal tissue development. (3) TEs are active epigenetic drivers of dedifferentiation, and in this role, they can critically contribute towards ageing and disease, including more aggressive cancer phenotypes.

## 2. Transposable Elements in the Human Genome

**Figure 1:**
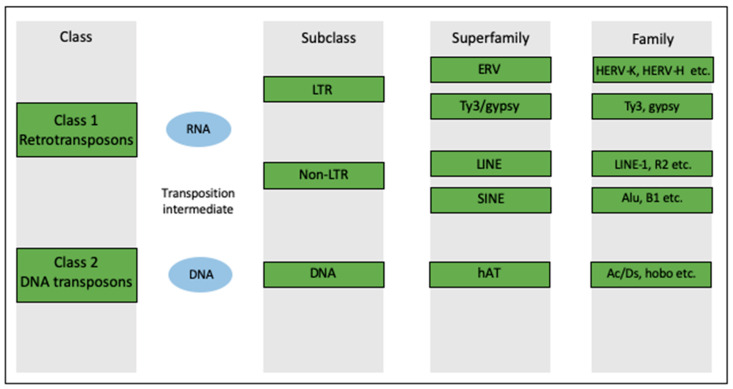
Classes and families of transposable elements within the human genome. Note that not all TE superfamilies and families are included in the figure.

TEs comprise two major groups, defined by the mechanisms they use to mobilise within the genome. DNA transposons use DNA intermediates to jump from one site into another within the genome. Alternatively, retrotransposons use RNA intermediates to propagate throughout the genome in a ‘copy/paste’ manner [1]. Each TE class can be further subdivided into subclasses based on integration mechanism and families based on their phylogeny (Figure 1). The widespread distribution of TEs across the genome suggests that repeated rounds of retrotransposition over evolutionary time have substantially expanded the size of the eukaryotic genome and contributed to the emergence of novel gene networks and regulatory elements [4]. However, due to the repetitive nature of these sequences, their analysis presents a formidable computational challenge [5]. This, together with the earlier misconception that transposable elements lack functional relevance, has left considerable uncertainty regarding the true extent of their contribution to genome evolution and regulation.

The human genome is primarily composed of four major TE orders: DNA transposons and three classes of retrotransposons—endogenous retroviral-derived long-terminal repeat (LTR), long interspersed nuclear elements (LINEs) and short interspersed nuclear elements (SINEs) [6]. These orders contain numerous families, and each may exist hundreds of times within the genome. Although TEs retain sequence similarity to once-active transposons, the vast majority of TEs (~99%) within the human genome have lost their ability to actively transpose [1]. This may imply the deprecation of transposition function; however, increasing evidence indicates that mammalian TEs have important roles beyond mobility. Recent studies have uncovered a diverse range of TE functions, including critical contributions to developmental regulation and mechanisms by which TEs can promote disease [7,8].

## 3. Factors Regulating Transposable Element Expression

TEs are largely transcriptionally silenced in healthy somatic tissues though DNA methylation—predominantly across TE sequences in bulk genomic regions rather than specifically at CpG (a cytosine followed by a guanine) islands—and through the recruitment of repressive chromatin marks [9,10]. This host-mediated repression of TE transcriptional activity in normal healthy somatic tissues [11,12] reflects their potential to cause dysfunction arising from their potential to move and propagate throughout the genome, including insertion into promoters or coding regions [6,7,8,9,10,11,12,13]. Additionally, the repetitive nature of these sequences can result in incorrect recombination events and lead to translocations, deletions and insertions. Loss of DNA methylation and subsequent TE activation have been proposed as a contributors to disease [14]. In general, DNA methylation at TE sequences is inversely correlated with transcription, and the evolutionary age of TEs can correspond to methylation levels during development [15]. More recent work in DNA methylation regulation indicates that promoter DNA methylation can result in paradoxical activation [16] and whether TEs are amenable to paradoxical activation is yet to be fully investigated.

During embryonic development, genome-wide demethylation following fertilisation coincides with a sharp increase in TE expression [17]. Some evolutionarily younger elements have been shown to maintain methylation during this genome-wide epigenetic reprogramming period, likely to prevent aberrant expression and transposition [7]. After implantation of the embryo, TE expression decreases in concordance with increased methylation. However, certain TE loci remain unmethylated, particularly in the extra-embryonic lineage. The placenta is well-known for TE expression and for harbouring unmethylated TE sequences, as reviewed recently [18].

## 4. Are Functional TEs Enriched in Developmental Tissues and Is Their Increased Post-Natal Methylation Level Developmentally Important?

TE transcript expression is enriched in early developmental tissues, suggesting potential functional roles. It remains unclear how the presence of TEs within transcripts influences their epigenetic regulation. TEs are epigenetically silenced in post-natal tissues to suppress their potentially deleterious effects [6,12]. However, many TEs within the human genome have lost their ability to actively transpose, and it is not clear whether they have diverged sufficiently to be distinct from loci that are capable of transposition [19,20]. Some studies suggest that there is overrepresentation of particular TE-derived transcript subfamilies in relation to their function as regulatory elements in certain tissues [21,22]. This suggests that TE-derived transcripts may facilitate the co-regulation of the resulting genes within a tissue type. Lower levels of DNA methylation in certain tissue types might potentially facilitate regulatory innovation involving TEs and could contribute to the abundance of functional TEs that are expressed in early human developmental tissues [23]. However, promoter or regional methylation is context-dependent and does not always strictly repress transcription.

TE-derived genes and regulatory elements are frequently expressed during early human development [18]. However, the enrichment of TE-derived transcripts in developmental tissues in comparison to post-natal tissues has not been conclusively studied. TE-derived gene expression and regulatory elements may be enriched during early human development in comparison to adult somatic tissues. For example, it has emerged that a high proportion of placental-enriched protein-coding genes are TE-derived when investigated by RepExpress analysis [24], which far exceeds the proportion of total protein-coding genes that have been discovered previously to have TE content. While there may be enrichment of TE-derived genes in the placenta, this is not conclusive. Further work is required to systematically characterise the proportion of TE-derived genes that are specific to other tissues.

Overall levels of TE transcript expression appear to be higher in human embryonic stem cells (hESCs) and in the human placenta compared to many somatic tissues, though the extent and functional consequences may vary across species and placental types [24]. Additional analyses are needed to address TE enrichment across different tissues, including utilising extensive datasets from a full range of somatic tissues. Importantly, TE expression is not always indicative of the presence of TE-derived genes and regulatory elements [19]. Examples exist of transposition events of evolutionarily young LINEs in somatic tissues, highlighting that TE expression can occur by multiple mechanisms [25]. Therefore, it will be necessary to carry out analysis in a full catalogue of somatic tissues to assess whether TE-derived genes and regulatory elements are indeed more prolific in early development. This analysis could utilise the wealth of publicly available RNA-Seq data or include more targeted approaches in a wide range of tissues. Moreover, it would be interesting to investigate expression in particular cell types, including those of the placenta. Given the heterogeneity of the placenta as a tissue, it could be highly informative to utilise cell sorting and analyse specific cell populations, particularly the invasive extra villous trophoblast cells.

Although much of the evidence linking TE-derived regulatory elements to gene expression is correlative, functional studies have begun to establish causal roles for some TE sequences. For example, Clustered Regularly Interspaced Short Palindromic Repeats interference (CRISPRi) and Clustered Regularly Interspaced Short Palindromic Repeats and CRISPR-associated protein 9 (CRISPR/Cas9) deletion of TE-derived enhancers in mouse stem cells have demonstrated that specific TE loci can act as bona fide enhancers, influencing gene regulatory networks during development [26]. Moreover, in human cancer cells, targeted CRISPRi and deletion of primate-specific LTR10 enhancers have been shown to reduce the expression of nearby genes such as *ATG12* and *XRCC4*, providing direct evidence of TE-mediated regulatory effects on tumour-relevant loci [27]. Although not all TEs are functional, a large number of functional TEs are enriched in early developmental tissues, particularly hESCs and the placenta [28,29]. The placenta was one of the first tissues in which TE-derived genes were identified [30]. Perhaps the first example of TE exaptation in the placenta was the discovery that envelope proteins (syncytins) are recruited to enable the fusion of trophoblast cells during early placentation [31]. This enables the fusion of syncytiotrophoblasts which is an essential part of placental development [31]. Additional examples of TE-derived promoters driving the expression of placental-enriched transcripts of protein-coding genes have also been identified [32]. Tissue-specific TE-long non-coding RNA (TE-lncRNA) associations were also identified by the same study, indicating that TEs may be recruited to facilitate tissue-specific regulatory networks involving long non-coding RNAs (lncRNAs).

More recently, further examples have revealed a role for TEs in other early human developmental tissue. Specifically, a significant proportion of octamer-binding transcription factor 4 (OCT4), homeobox transcription factor NANOG, and CCCTC-binding factor (CTCF) binding sites exist within TE sequences [33]. Interestingly, these binding sites tend to be those that are not conserved between mice and humans, suggesting that they could have facilitated regulatory evolution, particularly of stem cells, and might play a role in the maintenance of pluripotency. For instance, the human silencing hub complex (HUSH) also plays a key role in TE silencing, particularly in naïve pluripotent stem cells. HUSH binds to both endogenous retroviruses (ERVs) and LINE-1 (L1) elements and functions to repress evolutionarily young L1s. In contrast, in the mouse, KAP1 (KRAB-associated protein 1, also known as tripartite motif-containing protein 28, TRIM28) has a well-characterised role in transcriptional repression during early embryonic development. Deletion of KAP1 in ESCs results in upregulation of some TE sequences (ERVs), consistent with its role in recruiting SETDB1 to deposit H3K9me3 and repress these elements, indicating that KAP1 plays a key role in TE regulation within the early embryo [3]. This KAP1–SETDB1–H3K9me3 pathway provides a complementary mechanism to DNA methylation for stable TE silencing in early embryonic tissues [3]. Moreover, KAP1 and HUSH can function to co-repress some TEs in a tissue-specific manner [34]. While TE-derived genes and regulatory elements may influence development and cancer, functional validation is limited, and most mechanistic links remain correlative

A stem cell-specific class of long intergenic non-coding RNAs (lincRNAs) has also been identified, and expression of these lincRNAs is thought to be mediated by human endogenous retrovirus subfamily H (HERVH) elements [21]. Curiously, lincRNAs devoid of TEs are expressed at greater levels across different tissue types, particularly testis [21]. Altogether, this evidence supports but does not conclusively demonstrate that TE-derived genes and regulatory elements may be enriched in early human development. To draw conclusions, further analyses would be needed in a greater range of tissues. There are numerous questions remaining as to the roles these elements play in both development and disease.

## 5. Contribution of TEs to Early Development Through Regional Hypomethylation in Protein-Coding Genes and lncRNAs

TEs contribute to early development by facilitating context-dependent hypomethylation and the co-expression of protein-coding genes and lncRNAs, highlighting potential regulatory and evolutionary roles. It is unknown whether TEs possess a shared regulatory mechanism that facilitates coordinated hypomethylation, de-repression and co-expression of these genes in a developmentally enriched context, and which could shed light on the broader implications of TEs in development and the mechanism by which they are recruited. TEs within coding sequences or regulatory regions might have little or no functional consequence. However, TEs frequently harbour binding sites for key regulators of cell fate, particularly pluripotency factors and regulators of genomic architecture such as CTCF [33,35]. These binding sites become hypomethylated, allowing pluripotency factors to bind and potentially facilitating TE-associated regulation. Importantly, hypomethylation does not universally equate to transcriptional activation. Other examples of TE-derived protein-coding genes and lncRNAs that are critical regulators of development, such as Paternally Expressed Gene 10 (PEG10) [36,37] and Nuclear Paraspeckle Assembly Transcript 1 (NEAT1) [38], demonstrate that TE-derived genes can drive critical functions. Moreover, TEs can encode key functional domains within transcripts. The documented enrichment of TEs at the transcription start site (TSS) and 3′ regions of lncRNA and protein-coding gene transcripts reinforces the concept that TEs may encode some shared regulatory motif that facilitates context-dependent expression or interaction with other genomic factors, e.g., the LINE-1 five prime untranslated region (5′UTR) [39].

Supporting these mechanistic roles, studies in haematopoiesis have shown that CRISPR-Cas9-mediated deletion or epigenetic silencing of specific LTR-derived enhancers can lead to downregulation of nearby genes and impaired proliferation or survival in leukaemia cell lines, demonstrating that TE-derived enhancers can exert causal effects on gene regulation and cell phenotype [40]. These examples underscore that TEs can contribute functional regulatory elements that are not merely correlative but can directly influence developmental transcriptional programmes.

It remains unclear whether, from an evolutionary perspective, TE recruitment follows similar principles in protein-coding genes and lncRNAs, or whether these processes are mechanistically distinct. Given the higher degree of conservation of protein-coding genes in comparison to lncRNAs, and the often species- and tissue-specific function of lncRNAs [41], the mechanism may be distinct between the two. Specifically, investigating whether a TE evolves at the same time as the transcript, or becomes inserted into existing transcripts, would be an important distinction to make. It could be expected that TE-derived protein-coding genes arise due to insertions of TEs, which then function as alternate promoters or exons. However, it seems likely, given the huge contribution of TEs to lncRNAs and the often more recent evolution of lncRNAs, that TEs may have been more closely intertwined with lncRNAs and may have even facilitated their evolution [42]. Evidence in the broader literature also suggests a key contribution to regulatory elements such as enhancers [4,43]. Moreover, preliminary analyses of the evolutionary conservation of these elements suggest that the majority are either human- or primate-specific, suggesting a more recent evolutionary origin, which would also be interesting to explore further.

## 6. Why Is DNA Hypomethylation, Which Occurs in Early Embryonic Development, Important for TE Reactivation with Consequences for Ageing, Disease, Cancer and TE Evolution?

Early developmental hypomethylation facilitates TE activation, linking epigenetic regulation to consequences for development, disease, and evolution. DNA methylation is essential for repressing repetitive elements and plays critical roles in genomic imprinting and X chromosome inactivation [44]. DNA methylation silences transcription either by directly blocking the binding of transcriptional machinery or by recruiting repressive factors such as histone deacetylases that promote chromatin compaction. During early development, the genome is initially hypomethylated, after which de novo methyltransferases establish new methylation patterns in the embryo. Although methylation is reprogrammed genome-wide during cell differentiation, CpG islands typically remain unmethylated [45]. In gametogenesis, DNA methylation plays a key role in parental-specific imprinting [45,46]. Generally, promoter methylation is associated with stable gene silencing, especially at lineage-specific loci [47,48]. However, the relationship between DNA methylation and transcription is complex and promoter methylation alters the transcription of a subset of genes [49], while many others remain unaffected [16].

Similarly to the developing embryo, ESCs retain a unique epigenetic landscape that underpins their ability for self-renewal and pluripotency [50]. This is characterised by lower levels of DNA methylation, and a more open, accessible chromatin structure compared with differentiated cells [51]. Like ESCs, the placenta demonstrates a unique epigenetic landscape, with overall lower levels of DNA methylation compared to healthy somatic tissue [52]. Intriguingly, the placenta acquires methylation marks at the promoter regions of some genes, many of which are known as tumour suppressor genes [53]. The placenta is known to contain partially methylated domains (PMDs), an epigenetic feature that was previously thought to be an exclusive feature of malignant cells [54,55]. Overall, early developmental tissues appear to possess an epigenetic landscape that is less restricted than adult somatic tissue [56]. This open chromatin structure and reduced levels of genome-wide DNA methylation likely contribute to the unique functional properties of the placenta and pluripotent stem cells.

TEs have been recruited throughout evolution to function as genes and regulatory elements [57]. This is most apparent in early human developmental tissues (the placenta and ESCs) [14,58]. Previous research has speculated that the transient nature of the placenta, along with the hypomethylated state of the placental methylome, may have supported the evolution of functional TEs in the human placenta. The rapid evolution of the placenta, along with the huge array of diversity in structure and function of the placenta in eutherian mammals, could support the concept that the recruitment of TEs has been a key driver of placental evolution [4], although to date no studies have addressed this directly. Additional work has uncovered the important role of TEs in ESCs. ESCs have lower levels of DNA methylation and a higher developmental potential, which may have facilitated regulatory innovation, through the recruitment of TEs [59].

DNA methylation changes at TE-associated loci are not only characteristic of development but are also prevalent during ageing in humans [60] and in multiple diseases [61], such as cancer. While these observations suggest potential functional roles for TE-derived elements, most of the evidence remains correlative, and targeted perturbation experiments, such as CRISPR-mediated deletion or CRISPRi of TE-derived enhancers, are sparse. Traditionally, cancer genetics has focused primarily on mutational drivers of malignancy. However, the ‘epigenetic era’ has seen the rise in discoveries of epimutations ubiquitously associated with cancer [62]. This fast-growing field has revealed substantial epigenetic abnormalities in tumours that occur in combination with the mutational background of many cancers [63].

Mechanistic evidence in cancer models strengthens this view: in colorectal cancer cell lines, CRISPRi silencing and CRISPR/Cas9 deletion of LTR10 enhancers decreased the expression of genes, including *ATG12* and *XRCC4*, and altered responses (such as reduced viability after irradiation), illustrating direct effects of TE-derived regulatory elements on tumour phenotypes [27]. The functional enhancer activity of TE sequences has also been detected in other cancers, where TE subfamilies act as tissue-specific enhancers regulating gene expression, further supporting causal roles for TEs in oncogenic regulatory networks [64]. Importantly, emerging epigenome-editing approaches provide an additional and exciting strategy to dissect TE function with locus-specific precision. CRISPR/dCas9-based systems fused to DNA methyltransferases (e.g., DNMT3A) or demethylases (e.g., TET1 or TET2) can enable targeted editing of DNA methylation without altering underlying genomic sequence [65,66,67].

## 7. Shared Properties Between Early Developmental Tissues and Cancers

Loss of cellular identity and regression towards a less differentiated state are characteristic features of malignancy, which often correspond with the increased phenotypic plasticity and adaptability of cancer cells [68]. Many hallmark features of cancer mirror processes that are indispensable during early human development [69,70], such as proliferation, replicative immortality, angiogenesis, and a unique metabolism that supports these functions. Early development is also a pro-inflammatory state—similar to the tumour microenvironment [71,72]. Related to this, placental tissue can invade and modulate the immune system [73], and cancers often co-opt these same mechanisms [74,75]. In addition to these shared features, TE expression can directly engage innate immune-sensing pathways: TE-derived transcripts can form double-stranded RNA structures that mimic viral infection and are recognised by cytosolic RNA sensors such as retinoic acid-inducible gene I (*RIG-I*) and melanoma differentiation-associated gene 5 (*MDA5*), leading to type I interferon signalling and downstream antiviral responses in cancer cells, a process termed “viral mimicry” [76,77]. By contrast, some tumours may exploit TE expression to upregulate immune checkpoint molecules such as Programmed Death-Ligand 1 (PD-L1), contributing to immune evasion despite interferon activation [78]. TE activity can also yield novel peptides presented on major histocompatibility complex (MHC) molecules, generating neoantigens that may enhance adaptive anti-tumour immunity or represent targets for immunotherapy [79,80,81]. These distinct pathways—viral mimicry, immune evasion, and neoantigen formation—illustrate the multifaceted roles that TEs play in shaping cancer–immune interactions. Notably, species that have evolved to allow extensive invasion of the placenta show a higher incidence of epithelial cancers [82], and some cancers display enriched expression of placental genes [83].

The placental DNA methylome shares striking similarities with that of tumours [84]. During the first trimester, the human placenta exhibits large hypomethylated regions comparable in size and location to those of solid tumours [52,84], some of which become methylated as the pregnancy progresses. These genomic regions encompass genes involved in pathways central to both placental and cancer biology, such as epithelial to mesenchyme transition (EMT) as well as immune modulation and inflammation [84], suggesting that the shared functional properties of the placenta and cancer may be governed by similar epigenetic landscapes.

**Figure 2:**
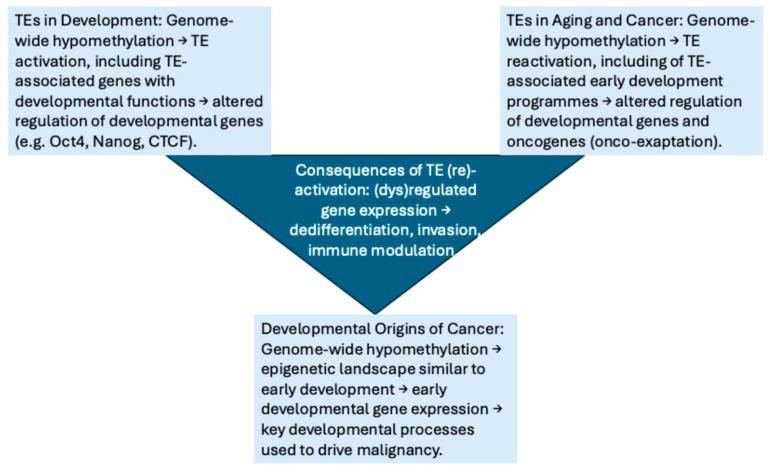
Summary diagram that depicts three currently distinct fields within the literature, suggesting that TE transcriptional activation might be associated with cancer dedifferentiation, and the developmental origins of cancer. Figure adapted from Lynch-Sutherland et al. (2020) [87].

Further studies have shown that epigenetic programming, which determines the identity of the extra-embryonic lineage as it diverges from the embryo proper, resembles the somatic transition to cancer [85]. Parallels between somatic reprogramming to induced pluripotent stem cells (iPSCs) and pathological reprogramming in cancer further highlighted the substantial overlap between development and malignancy [69]. A recent study of human somatic cell reprogramming to a pluripotent state identified a role for transcription factors during this process [86]. They also showed that a subpopulation of cells enters a trophoblast-like state during reprogramming. When isolated for further analysis, the trophoblast-like stem cells could be induced to differentiate along the trophoblast lineage. This work provides a compelling potential mechanism for the reactivation of placental genes during oncogenic reprogramming [86]. Taken together, these findings demonstrate substantial similarities between early development and cancer, and they support the concept that the reactivation of early developmental genes likely facilitates cancer cells to recapitulate the early developmental state with functional relevance for tumour progression (Figure 2).

## 8. Molecular Evidence for Altered Methylation of TEs in Cancer: Is TE Transcriptional Activation Associated with Cancer Dedifferentiation?

Disruption of the methylation landscape is a hallmark feature of cancer cells, largely characterised by a reduction in global DNA methylation levels (hypomethylation), which has been functionally linked to genomic instability [88]. Interestingly, despite a reduction in global DNA methylation levels, cancer cells show consistent hypermethylation of some CpG islands [89]. These regions usually overlap with the promoter regions of certain genes, such as tumour suppressor genes, which has been shown to be responsible for their transcriptional silencing [88,89,90]. Whilst this striking discordant methylation pattern (when compared to healthy somatic tissue) is observed consistently across multiple cancer types, the underlying mechanisms remain elusive. It remains unknown as to how de novo methylation is acquired preferentially at promoter regions of tumour suppressor genes, whilst in some cases oncogene promoters simultaneously lose DNA hypomethylation. Nevertheless, the potential to reverse epigenetic alterations makes them promising potential targets of novel epigenetic therapies. The discovery that ESCs may result in tumour formation when transplanted into adult tissue was fundamental to the field of cancer epigenetics [91].

While DNA methylation regulates TE-derived genes, it is unclear whether they are initially demethylated in early development and later methylated in somatic tissues, or if methylation is established early and subsequently lost along placental lineages. It would be of interest to survey methylation in a range of different stages of early human development to assess how dynamically the methylation of TE candidate genes changes throughout development. Recent work [24] shows that developmental-enriched, TE-derived genes and regulatory elements become transcriptionally reactivated in cancer, associated with altered TE methylation levels [87,92]. Some TE-derived genes have been shown to be activated across multiple different cancer types, associated with reduced global methylation levels [24]. Whether reduced global methylation in cancer leads to dedifferentiation, or whether TE expression is a causal contributor or a consequence of these processes, is unknown.

Global DNA hypomethylation can lead to de-repression of normally silenced genomic regions, including immune-related loci, resulting in aberrant immune gene expression. Such dysregulated immune signalling may be exploited by cancer cells to remodel and evade host immunity, one of the defining hallmarks of cancer [93,94]. Interestingly, TE expression has been linked to both immune suppression and increased antigenicity of tumours [78,79,80]. Further work is needed to tease out specifically which TEs are associated with these two conflicting functions. Importantly, this would be of significant clinical utility given the significance of immune evasion in tumour progression. There is striking variability in patient response to current immunotherapies, and current prediction of response is lacking. It is possible that some TE-derived genes may function as tumour-associated neoantigens and thus may facilitate antigenicity. However, some may also be associated with immune evasion and/or dedifferentiation as an escape route to immunotherapies. Thus, establishing the full complement of functions that TEs and TE-derived genes can play in immune modulation in the context could be hugely illuminating to the field of immunotherapy.

Given the abundance of TEs within the genome, relatively few seem to have evolved to have acquired regulatory or coding functions. This raises the question as to what determines which TEs are recruited to perform key functions. It is likely that the local genomic architecture plays a key role in this process, and therefore TEs within active, euchromatic regions and close to transcriptionally active genes may be predisposed to ‘domestication’. However, this would predict that TEs in the vicinity of universally transcribed genes may be functionally enriched. Although this idea has not been investigated directly, current evidence suggests that TE-derived genes show more tissue-restricted expression patterns [17,21]. With respect to the literature implicating TEs in cancer, it is important to contemplate whether TEs themselves predispose to cancer, or whether they just contribute disproportionately to developmental-enriched genes and regulatory elements and thus facilitate processes that contribute to cancer. To this end, when considering the mechanism for the reactivation of developmental TE-derived genes in cancer, it should be queried as to whether TEs are ubiquitously reactivated in cancer (resulting in the reactivation of TE-derived genes and regulatory elements), or if TE expression occurs as a result of the re-establishment of an early developmental epigenetic landscape and the resulting reactivation of developmental genes, which are enriched for TE sequences.

It is crucial to define the context, including timing and tissue and cell type, in which developmental TE-derived genes are reactivated in cancer. Reactivation of placental-enriched TE-derived genes appears to occur across several cancer types [95,96]. Considering the invasive nature of placenta, such reactivation may also contribute to tumour invasiveness. Similarly, expression of prognostic enhancer elements shared between hESCs, placenta, and tumours suggests that developmentally derived enhancers may hold prognostic or therapeutic value, about half of which appear to be TE-derived [4,43,58]. However, only a small subset of intergenic and intronic TEs have been studied for enhancer activity, underscoring the need for functional validation studies [26]. Confirming enhancer function and identifying their target genes will be critical, as many harbour motifs for key pluripotency regulators such as OCT4, implying shared regulatory networks between development and cancer.

Studying dedifferentiation in the context of induced pluripotent stem cells (iPSCs) has enabled insights into the epigenetic signatures of dedifferentiation and highlighted the potential for cancer cells to dedifferentiate during tumour progression [69]. However, the mechanisms which induce dedifferentiation within the cancer microenvironment remain elusive, as does the complete picture of the genetic and epigenetic signatures [62]. Incomplete reprogramming of somatic cells to iPSCs can drive malignant transformation, supporting the concept that epigenetic signatures of reprogramming can drive cancer, even in the absence of the mutation profile, which is often characteristic of cancer genomes [97]. The presence of cells that exist in a less differentiated state and have unparalleled replicative potential is considered a common feature of many cancers. These cells have been termed cancer stem cells, and whilst their existence is widely accepted, it remains contentious as to the frequency at which they exist, along with their origins [91,98,99]. Specifically, it remains unclear whether tumours dedifferentiate to acquire stem-cell-like properties and increased phenotypic plasticity or if in fact some tumours are derived from existing stem cell populations that have procured genetic and epigenetic alterations to drive dysfunction.

The observation that some cancers can be induced to differentiate in response to certain factors supports the suggestion that dedifferentiation of some cancer cells may occur, giving rise to cancer stem cells and facilitating phenotypic plasticity [100,101]. However, other studies have shown that tissues with a prevalent stem cell population may have an increased incidence of malignancy, providing circumstantial evidence of the latter [71,102]. Furthermore, it has been shown that global epigenetic changes can precede any genetic mutational events in some solid tumours [103]. Perhaps the most captivating argument towards the epigenetic progenitor origin for cancer is in mouse models; when a malignant cell nucleus (melanoma—a cancer characterised by high mutational burden) is cloned, it can differentiate into a normal healthy embryo, indicating that given the correct microenvironment, tumours can be reprogrammed to traverse a normal developmental trajectory [104]. Nevertheless, there is now conclusive evidence suggesting a critical role for epigenetic aberrations in facilitating malignant transformation. Moreover, regardless of how cancers obtain a less differentiated epigenetic landscape, deciphering how to reprogramme this in vivo will have huge implications clinically.

Intriguingly, some cancers have been shown to express not just early developmental genes derived from the embryo but also those that are exclusive to the extra-embryonic lineage, including the placenta [95,105]. While reactivation of some placental-enriched TE-derived genes occurs in cancers, this should not be interpreted as implying that all placental functions are recapitulated in tumours. The placenta possesses unique functional properties that are exclusive to placentation and are not seen in any other healthy tissue, which has been reviewed elsewhere [82,106].

## 9. What Roles Do TEs and Their Hypomethylation State Play in Cancer?

Onco-exaptation events have been previously identified in cancers [107] which has revealed that some onco-exaptation events may not be novel to cancer, but they may occur as the result of reactivation of developmental-enriched TE–oncogene regulatory relationships. The role of onco-exaptation in cancer has recently been reviewed [108].

There are many potential implications of TE expression in cancer. For example, TEs are highly enriched in early development and cancer, and they have the potential to be used as biomarkers. The expression of germline and placental genes has been implicated as a marker of more aggressive and metastasis-prone tumours in multiple different tissues, showing that they may have utility in this context [109].

Functional studies could establish whether TE-associated genes promote malignancy. Their specificity to early developmental tissues and cancer makes them attractive therapeutic targets, as they are not expressed in healthy adult tissues. Further analysis would be required to establish absolute specificity to development and cancer. This would require the investigation of a full complement of healthy somatic tissues, and the analysis of single-cell sequencing data. TEs have been shown to be variably expressed in different cell types of the same tissues, highlighting the need for such investigation [110]. Moreover, the dedifferentiation of cancer cells is associated with invasion and metastasis. Therefore, the identification and targeting of genes that contribute to dedifferentiation may be utilised to prevent tumour progression. Dedifferentiation and expression of early developmental genes are associated with more aggressive and deadly cancers, and have been linked to treatment resistance [109,111,112]. Currently, the only ultimately effective treatment with the greatest potential for preventing metastasis from occurring is the complete resection of the primary tumour by surgery. Identifying TE-derived genes that contribute to dedifferentiation may help to prevent invasion and metastasis through either early diagnosis and/or treatment, which overall would improve patient outcomes. If TE-derived genes play a key role in dedifferentiation, perhaps they may be biomarkers against which the development of specifically targeted therapies could help to reduce cancer-related deaths.

It has yet to be established whether cancer stem cells are derived from somatic cells that have dedifferentiated to acquire stem cell-like traits and increased oncogenic properties, or whether these cells are derived from tissue stem cells that exist in a less differentiated state. Some cancers cells can be induced to dedifferentiate in vitro in response to either inflammation or immunotherapy [112,113,114]. In melanoma, partial reprogramming can induce cells to switch to an invasive phenotype [100]. However, the key question is how to address dedifferentiation clinically, in order to target cancer stem cells therapeutically. Delineating the interplay between epigenetics, dedifferentiation, phenotypic plasticity, EMT cancer stem cells and TEs are a crucial next step in the field.

Some studies have carried out drug screens to identify possible therapies that are able to target less well-differentiated cells [113]. Identifying sensitivities that are specific to this population of cells would be crucial in targeting them therapeutically. It is likely that a combination approach would be optimal, one which enables the targeting of cancer stem cells in conjunction with targeting overall tumour growth. Given the evidence for phenotypic plasticity and phenotype switching between different states in cancers, a therapeutic approach that takes this into account may help to optimise treatment outcomes for patients. Therefore, if TEs potentially drive a dedifferentiated or invasive state, then therapeutic targeting of TEs could be beneficial.

## 10. Do TEs and Their Hypomethylation State Contribute as Epigenetic Drivers of Cancer?

The existence of TEs within genes and regulatory elements that are expressed specifically in early development and cancer is a fascinating phenomenon. However, it remains to be determined whether these functional TEs are merely enriched within developmental genes and their regulatory elements, which consequently become reactivated in cancers to regulate the same processes, or whether TE-derived genes are preferentially reactivated in cancer in association with a shared regulatory mechanism. Previous studies have implicated TEs as becoming preferentially demethylated in some cancers [88,115,116]. However, it has not been established whether this primarily occurs due to their methylated state in healthy somatic tissue, and their abundance within the genome that is accountable for this phenomenon. A recent study showed that locus-specific transcriptional regulation of TEs by p53 binding is evident in colorectal cancer cells versus normal cells, suggesting there is a potential role of chromatin context in TE regulation [117]. Further work analysing what drives demethylation, as well as altered chromatin contexts of TEs in cancer, will be needed to assess whether epigenetic alterations are targeted specifically to these elements, which could possibly be due to their high sequence homology. Establishing whether it is developmental genes (in general) or developmental TE-derived genes that become reactivated in cancer will require investigations into the proportion of developmental TE-derived genes and regulatory elements that become reactivated in cancer versus those developmental genes and regulatory elements not derived from TEs that become reactivated. There is certainly mounting evidence suggesting that TE-derived genes might be more tightly regulated and have more tissue-restricted expression patterns [21], which in some respect makes their recurrent reactivation in cancer surprising.

It remains unclear whether TE expression drives tumour dedifferentiation or occurs as a consequence (i.e., whether TEs are drivers or passengers in these processes). TE-derived enhancers reactivated in melanoma cell lines suggest broader regulatory functions beyond individual genes [24]. Enhancer elements are known to play a vital role in regulating the transcriptome, particularly super enhancers, which can be key determinants of cell fate [118,119]. The identification of novel TE-derived enhancer elements that are specific to early development and cancer, along with the discovery that some prognostic enhancers are also TE-derived and developmentally expressed, is compelling. Additionally, the existence of many hESC-specific binding sites for CTCF, OCT4 and CTCF [33], and the role of TEs in demarcating topologically associated domains in hESCs [35], supports the potential of a highly influential role for TEs in driving dedifferentiation in cancer. Is it possible that “development-like” epigenetic landscapes in cancer essentially recapitulate an early embryonic developmental landscape of a normal cell, as has been suggested in Waddington Landscape models of epigenetic remodelling in cancer [120]. Decrypting local and genome-wide regulatory mechanisms of TEs in both development and cancer will be essential, and a critical part of this puzzle, in terms of realising the potential of TEs as regulators of development and disease. A key outstanding question is whether TEs act as drivers of dedifferentiation and tumorigenesis, directly influencing gene regulatory networks, or as passengers, becoming reactivated as a consequence of broader epigenetic remodelling in early developmental-like states. Evidence to date is largely correlative, though some locus-specific functional studies suggest potential driver roles for specific TE-derived elements. Further work, including CRISPR perturbation and single-cell analysis, will be required to distinguish these models and clarify the causal contributions of TEs to dedifferentiation and cancer progression.

## 11. Future Directions

The challenges and opportunities presented here raise many questions. Some of the relevant questions are listed in Table 1. 

An obvious next step would be the functional characterisation of genes and regulatory elements to decipher their roles in both early development and cancer. For example, antisense oligonucleotide (ASO)-mediated knockdown experiments of candidate genes could be undertaken, in combination with invasion, migration and spheroid assays to assess whether these genes have a functional role in cancer cell lines. ASOs are showing promise in the clinic as a therapeutic approach to target mRNAs and lncRNAs [121]. Multiple ASOs are currently undergoing clinical trials for various diseases (including cancer) and are showing encouraging results. Thus, the identification of novel targets for ASO-mediated therapies has the potential to greatly improve cancer treatment through enabling a more personalised approach. Recently, TEs were suggested as novel potential therapeutic targets for Poly (ADP-ribose) polymerase inhibitor (PARPi)-induced synthetic lethality in polycomb group (PcG)-mutated blood cancers [122], supporting the idea that TEs could be suitable targets in cancer using ASO-mediated therapies. Additionally, CRISPRi screens could be used to identify genes that are essential for cancer cell survival. Ultimately, this may help to establish whether TE-derived genes underlie some of the shared properties of early development and cancer, and whether targeting them could have clinical utility. Analysis of single-cell sequencing data may be used to confirm the specificity of the expression of TE-derived genes in early development, in which case these genes could then enable the targeting of cancer cells specifically, thus minimising harmful side effects for patients.

It would also be interesting to establish the roles of the TE itself within the context of associated transcripts. The noted enrichment at the TSS and the 3′ ends of transcripts supports that TEs may encode key regulatory domains that mediate transcription and/or posttranscriptional regulation. Furthermore, investigation of whether the presence of TEs enables co-regulation of these elements would be highly informative. Indeed, the identification of classes of tissue-specific lncRNAs (in hESCs and the placenta), that are enriched for a certain TE subfamily at their TSS, suggests that TEs may facilitate tissue-specific co-regulation of transcripts [74]. Additional characterisation of the regulatory mechanisms that function to mediate TE expression should also be prioritised as this could shed light on the mechanisms permitting activation of these genes/elements in early development and in cancer. Future research could also focus on finding a balance between the investigation of TEs at both the local and genome-wide level. For many years, studies have been biassed towards grouping TEs into subfamilies for analysis, which has potentially lost some biological significance, particularly if TEs are regulated based on their genomic context rather than due to the high homology between subfamilies.

It is becoming clear that a more integrative approach within the field of epigenetics is needed. Although DNA methylation has been a key focus of several cancer-related TE studies to date, it is increasingly apparent that epigenetic mechanisms do not function in isolation; therefore, studies could benefit from the investigation of both a wider range of epigenetic mechanisms, along with assessment of a broader range of tissues. Development is a highly dynamic process and therefore investigating only brief snapshots is limited and does not allow for a complete picture. Surveying a large selection of TEs in a broader range of developmental stages and tissues would be highly informative and would help to decipher the mechanism which facilitates the restricted expression of these TEs in healthy somatic tissues, yet permits their reactivation in tumours.

## 12. Concluding Remarks

In this article, we have reviewed TEs, their epigenetic regulation (particularly by DNA methylation) and their roles in normal development and cancer. We propose that TEs may act as oncogenic drivers by reactivating developmental programmes shared between these tissues. Understanding this relationship could reveal key molecular parallels between early human development and tumorigenesis. If robust validation and functional studies are performed, TE-driven pathways may offer novel therapeutic opportunities to target cancer-specific phenotypes while sparing normal tissues. Although TEs are established regulators of early development, their potential oncogenic driver role remains to be further defined. This challenge is compounded by inter- and intra-tumour heterogeneity and analytical challenges which obscures the distinction between driver and passenger events. Comprehensive functional characterisation of TE-associated genes and their epigenetic control could uncover new therapeutic targets and foster a more integrated view of developmental and cancer biology, ultimately enhancing therapeutic options and advancing clinical outcomes.

## Figures and Tables

**Table 1:** Outstanding questions regarding transposable elements.

Transposable Elements Outstanding Questions	
Q1	Is transposable element (TE) activation a causal contributor to tumour dedifferentiation and progression, or a consequence of broader epigenetic reprogramming?
Q2	Is TE reactivation an early event in transformation, or does it emerge during progression, invasion, and metastasis?
Q3	Why do only a small subset of TE loci become exapted as functional enhancers, promoters, or coding elements in development and cancer?
Q4	Does TE reactivation in cancer arise from locus-specific epigenetic regulation, or simply from a process involving global genome-wide DNA hypomethylation?
Q5	How do specific TE families differentially contribute to viral mimicry, immune evasion, or neoantigen formation?
Q6	Are TE associated genes or enhancers sufficiently tumour-specific to serve as biomarkers or selective therapeutic targets?

## Data Availability

No new data were created or analyzed in this study. Data sharing is not applicable to this article.
